# *Bifidobacterium longum* CECT 7894 Improves the Efficacy of Infliximab for DSS-Induced Colitis *via* Regulating the Gut Microbiota and Bile Acid Metabolism

**DOI:** 10.3389/fphar.2022.902337

**Published:** 2022-08-01

**Authors:** Fangfei Xiao, Fang Dong, Xiaolu Li, Youran Li, Guangjun Yu, Zhanju Liu, Yizhong Wang, Ting Zhang

**Affiliations:** ^1^ Department of Gastroenterology, Hepatology and Nutrition, Shanghai Children’s Hospital, School of Medicine, Shanghai Jiao Tong University, Shanghai, China; ^2^ Gut Microbiota and Metabolic Research Center, Institute of Pediatric Infection, Immunity and Critical Care Medicine, School of Medicine, Shanghai Jiao Tong University, Shanghai, China; ^3^ Department of Gastroenterology, The Shanghai Tenth People’s Hospital of Tongji University, Shanghai, China

**Keywords:** inflammatory bowel diseases, *Bifidobacterium longum*, infliximab, gut microbiota, bile acids

## Abstract

**Background:** Recent evidence suggests that the changes in gut microbiota and its metabolites could predict the clinical response of anti-tumor necrosis factor (TNF) agents, such as infliximab (IFX). However, whether manipulation of the gut microbiota can enhance the efficacy of anti-TNF agents remains unclear. Here, we aim to evaluate the effect of a probiotic strain, *Bifidobacterium longum* (*B. longum*) CECT 7894, on IFX efficacy for dextran sulfate sodium (DSS)-induced colitis in mice and attempt to explore the potential involved mechanisms.

**Methods:** C57BL/6 mice were treated with phosphate-buffered saline (PBS) or *B. longum* CECT 7894 (5 × 10^8^ CFU/day) once daily by gavage for 5 days and subsequently induced acute colitis by 3% (w/v) DSS in drinking water. The efficacies of IFX combined with or without *B. longum* CECT 7894 were assessed by weight loss, fecal consistency, colon length, and histopathological changes. Immunohistochemistry (IHC) was used to examine the expression of tight junction proteins (TJPs) in colonic tissues. The microbiota composition was characterized through 16 S rRNA gene sequencing. Fecal bile acids (BAs) levels were analyzed by targeted metabolomics.

**Results:** *B. longum* CECT 7894 improved the efficacy of IFX for DSS-induced colitis as evidenced by decreased weight loss, disease activity index (DAI) scores, colon length shortening, histological damage, increased ZO-1, and Occludin expressions as compared with mice that received IFX only. *B. longum* CECT 7894 modified the composition and structure of the gut microbiota community in DSS-induced colitis mice. *B. longum* CECT 7894 increased the relative abundances of genera *Bifidobacterium*, *Blautia*, *Butyricicoccus*, *Clostridium*, *Coprococcus*, *Gemmiger*, and *Parabacterioides*, and reduced the relative abundances of bacteria genera *Enterococcus* and *Pseudomonas*. Furthermore, *B. longum* CECT 7894 changed the BAs metabolism by increasing the abundance of secondary BAs, such as *a*-MCA, *ß*-MCA, LCA, CDCA, UDCA, HCA, isoLCA, isoalloLCA. The covariance analysis revealed the upregulated secondary BAs were positively associated with the increased abundance of bacteria that contained bile salt hydrolases (BSH) and 7α-dehydroxylases genes.

**Conclusion:** *B. longum* CECT 7894 improved the efficacy of IFX for DSS-induced colitis *via* regulating the gut microbiota composition and bile acid metabolism. Probiotics supplementation may provide a possibility to improve the clinical response of anti-TNF agents in IBD management.

## Introduction

Inflammatory bowel disease (IBD) is a group of chronic and relapsing gastrointestinal inflammatory disorders caused by complex and multifactorial etiologies (Khor et al., 2011). Patients with IBD are usually complicated by various manifestations that significantly impair their quality of life, including recurrent diarrhea, abdominal pain, fistulae, stenoses, abscesses, perianal lesions, and extraintestinal symptoms (Nikolaus and Schreiber, 2007). Although multiple drugs have been applied in the clinical treatment of IBD, such as 5-Aminosalicylates (5-ASAs), corticosteroids, immunosuppressive drugs, and biological agents (e.g., anti-tumor necrosis factor (TNF) agents), there are no curative therapies for IBD (Torres et al., 2020).

Anti-TNF therapy has become the backbone of treatment since its first introduction in the management of IBD in 1993 (Derkx et al., 1993). The mechanism of action of anti-TNF therapy in IBD is mainly attributed to the neutralization of soluble TNF (Levin et al., 2016). Anti-TNF agents, such as infliximab (IFX), are widely used for moderate to severe IBD patients for achieving a high clinical response rate and obtaining mucosal healing. However, nearly 30% of IBD patients do not have an initial clinical response to anti-TNF agents, and less than 50% of them could achieve mucosal healing eventually (Colombel et al., 2010; Roda et al., 2016). It is of significant clinical value to improve the efficacy of anti-TNF agents by both identifying biomarkers for predicting the clinical response and exploring combination therapy with other drugs in IBD. For instance, age, body mass index (BMI), duration of disease, fecal calprotectin, serological antibodies, gut microbiota, and metabolomics were shown to be associated with the response of anti-TNF therapies in IBD patients (Ding et al., 2016; Aden et al., 2019; Ding et al., 2020). It has been shown that IFX and azathioprine combination therapy was superior to achieve corticosteroid-free clinical remission than monotherapy in IBD (Colombel et al., 2010).

Recent compelling evidence has demonstrated that disturbed gut microbiota correspond with altered microbial metabolic functions and are involved in the pathogenesis of IBD (Glassner et al., 2020; Lavelle and Sokol, 2020). Compared to healthy subjects, IBD patients present a reduced abundance of phyla *Firmicutes* and *Bacteroidetes*, while an expansion of potentially pathogenic bacteria including *Escherichia coli*, *Klebsiella pneumonia*, and *Neisseriaceae* (Jacobs et al., 2016; Lloyd-Price et al., 2019). Metabolomics studies have revealed microbial metabolite alterations in patients with IBD, such as fecal bile acids (BAs) (Duboc et al., 2013; Yang Z. H et al., 2021). The increased levels of primary BAs and decreased levels of secondary BAs were observed in IBD patients (Duboc et al., 2013; Yang Z. H et al., 2021). Thus, microbial-based therapies for restoration of the gut microbial composition and microbial metabolisms may provide new options for either IBD monotherapy or combination therapies, such as fecal microbiota transplantation (FMT) and probiotic supplementation (Oka and Sartor, 2020). Currently, whether microbial-based therapies can improve the efficacy of anti-TNF treatment remains undetermined.

We previously showed that the relative abundance of *Bifidobacterium* was reduced in pediatric Crohn’s disease (CD) patients (Wang et al., 2018; Wang et al., 2021). *Bifidobacterium* species, such as *Bifidobacterium longum* (*B. longum*), contain bile salt hydrolase (BSH) genes that play a critical role in BA metabolism (Ruiz et al., 2021). In this study, we aimed to investigate the effect of a probiotic strain isolated from the stool of a healthy child, *B. longum* CECT 7894 (Santas et al., 2015), on the efficacy of IFX for attenuating intestinal inflammation in DSS-induced colitis mice. We further attempted to explore the potential mechanisms involved by analyzing the changes in the gut microbiota community and BAs metabolism.

## Materials and Methods

### *B. longum* CECT 7894 Preparation

*B. longum* CECT 7894 strain obtained from Dipro AB-7894 Drops (AB-Biotics. S/A, Barcelona, Spain) was recovered in Man Rogosa Sharpe (MRS, Sigma-Aldrich, United States) agar plate for 24 h at 37°C, anaerobically. Bacteria amplified from a single colony in an anaerobic jar in MRS broth were harvested by centrifugation at 4,000 rpm for 10 min at 4°C, then washed 3 times with sterile phosphate-buffered saline (PBS) and resuspended with sterile PBS to a concentration of 2.5 × 10^9^ colony-forming units (CFU)/mL for gavage.

### Animals

C57BL/6 mice (6–8 weeks of age, female, weighing 18.0 ± 2.9 g) were purchased from Hangzhou Ziyuan Experimental Animal Technology Co. Ltd. (Hangzhou, China). Mice were housed under specific pathogen-free (SPF) conditions with 12 h light/dark cycle at 22°C and given free access to food and water. All animal experimental procedures involved in the study were approved by the Animal Ethics Committee of Shanghai Children’s Hospital (SHCH-IACUC-2021-XMSB-41).

### Probiotic Supplementation, Colitis Induction, and IFX Infusion

**FIGURE 1:**
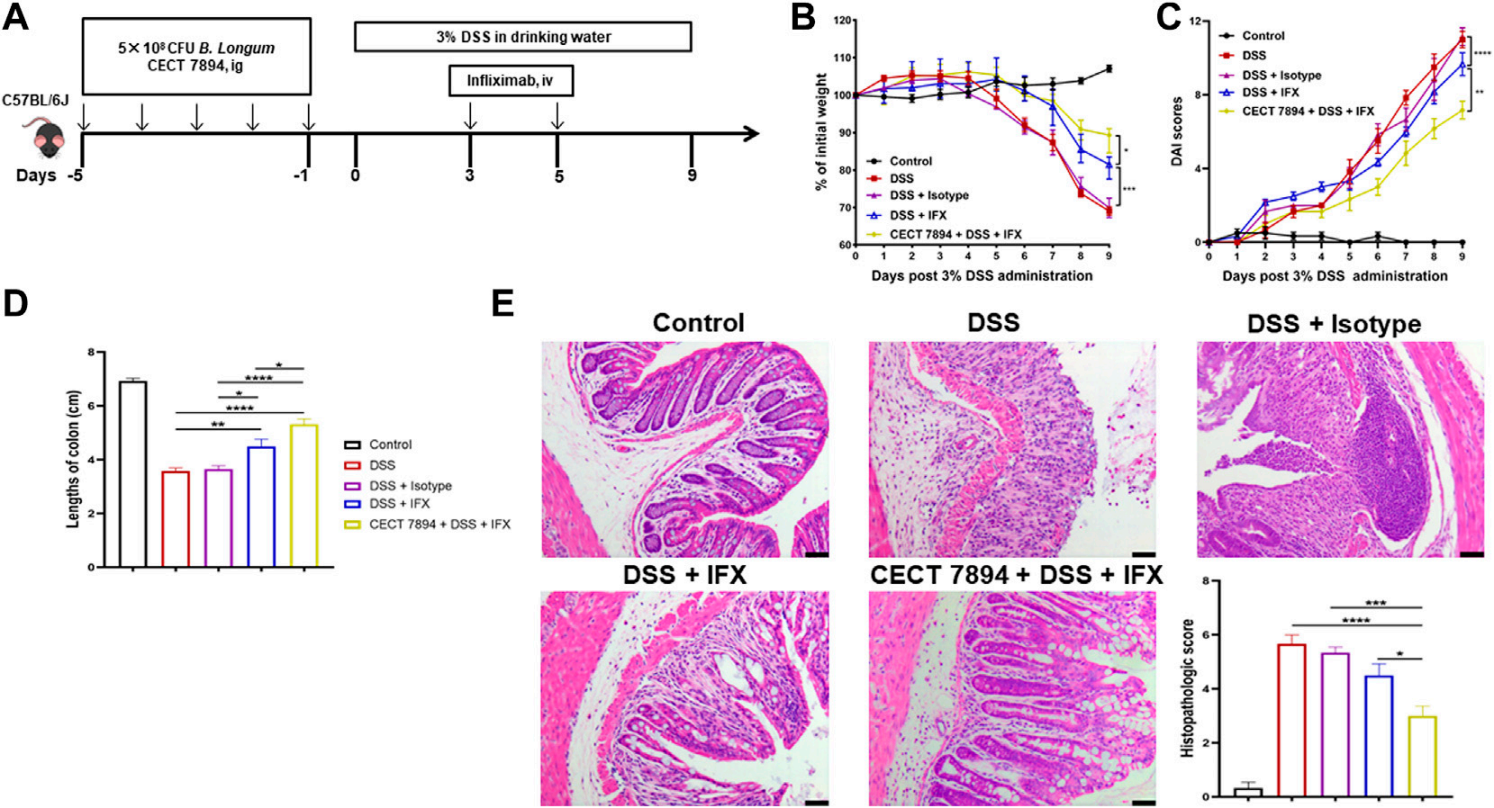
*B. longum* CECT7894 improves the efficacy of IFX for DSS-induced colitis in mice. **(A)** Diagram illustrating the mouse model of colitis employed in this study. **(B)** Body weight changes. **(C)** The disease activity index (DAI) assessment. **(D)** Colon length at day 9. **(E)** H&E staining analysis and histology score. Scale bar represents 25 µm in each image. Data were presented as mean ± SEM (*n* = 6 per group). Statistical significance was assessed by using one-way ANOVA followed by Tukey’s test. *p ≤ 0.05, **p ≤ 0.01, ***p ≤ 0.001, ****p ≤ 0.0001.

After a week of acclimation, mice were randomly assigned to five experimental groups containing 6 animals: the control group, DSS group, DSS + Isotype group, DSS + IFX group, and *B. longum* CECT 7894 + DSS + IFX group. For probiotic pre-treatment, each mouse in the *B. longum* CECT 7894 + DSS + IFX group was given 200 μL *B longum* CECT 7894 solution (5 × 10^8^ CFU) by oral gavage once per day for 5 days, while the mice in other groups received 200 μL PBS by oral gavage instead. After 5 days of pre-treatment, the mice were administered 3% (w/v) DSS in their daily drinking water for 9 days to induce acute colitis. Mice in the control group were given sterile water. For IFX treatment, mice were injected with 100 μL IFX (5 mg/kg) or isotype at day 3 and day 5 (Figure 1A). Body weight, fecal consistency, and the presence of blood in the feces were monitored from days 1–9 in order to calculate the disease activity index (DAI) as previously described (Wirtz et al., 2017).

### Histological Assessment

A 5 mm distal colon tissue was fixed by 4% paraformaldehyde, embedded in paraffin, and sections of paraffin were stained with hematoxylin and eosin (H&E). The severity of colitis was assessed based on a 0 to 4-point scale based on the previously described protocols (Wirtz et al., 2017; Spalinger et al., 2019). The assessment included 2 parameters, respectively: 1) inflammatory cell infiltration (0 = none, 1 = mucosa, 2 = mucosa and submucosa, and 3 = transmural); 2) tissue damage (0 = none, 1 = isolated focal epithelial damage, 2 = mucosal erosions and ulcerations, and 3 = extensive damage deep into the bowel wall).

### Immunohistochemistry Assay

Immunohistochemistry (IHC) was performed in murine distal colon sections to investigate the expression of tight junction proteins (ZO-1 and Occludin) and farnesoid-X receptor (FXR). The colon tissues were immersed in 4% paraformaldehyde for 24 h and then embedded in paraffin. The tissues were sectioned into slices (6 μm). Antigen retrieval was performed by boiling slides for 20 min in EDTA citrate buffer (pH = 7.8). The sections were incubated with antibodies specific for ZO-1 (Bioworld Technology; BS9802M; 1:100), Occludin (Bioworld Technology; BS72035; 1:100), and FXR (abcam; ab235094; 1:100) at 4°C overnight. After washing with PBS/Tween20 buffer (pH = 7.6), the sections were detected using an anti-rabbit secondary antibody for half an hour at room temperature, respectively. Reactions were revealed by a DAB chromogenic reagent kit. Then the intensity of staining was observed by a microscope (Leica DMi8) and analyzed by ImageJ Java.

### Fecal Microbiota Analysis

Genomic DNA of each collected feces was extracted using the QIAamp DNA Stool Mini Kit (Qiagen, Hilden, Germany) according to the manufacturer’s instructions. The 16 S universal primers 341 F (5′-CCT​ACG​GGA​GGC​AGC​AG-3′) and 806 R (5′-GGACTACHVGGGTWTCTAAT-3′) were used to amplify the V3-V4 hypervariable regions of the eubacterial 16 S rRNA gene and sequenced on Illumina NovaSeq platform (Illumina, United States). Raw sequence data were quality control checked using fastp (version 0.20.0) and merged by FLASH (version 1.2.7). Chimeric sequences were removed using data2, which generated unique amplicon sequence variants (ASVs) and a feature table (Callahan et al., 2016). Further analysis was conducted in a data curation pipeline implemented in the QIIME2 (version 2020.2) platform (Bolyen et al., 2019). The *alpha* diversity of the microbial community was conducted by the index of observed species, Chao1, abundance-based coverage estimator (ACE), Shannon, and Simpson, and the *beta* diversity was calculated using Bray-Curtis distance and visualized by principal coordinate analysis (PCoA). Kruskal–Wallis test was applied to assess the relative abundance of taxa differences between groups. The significant differences in the gut microbiome structure were estimated by permutational multivariate analysis of variance (PERMANOVA) analysis. The linear discriminant analysis (LDA) effect size (LEfSe) method was performed to characterize the statistical significance and biological relevance of enriched microbiota taxa in different groups. Functional profiles of metabolic pathways enrichment analysis were imputed to PICRUSt v.2.4.1 (Phylogenetic Investigation of Communities by Reconstruction of Unobserved States) software using the Kyoto Encyclopedia of Genes and Genomes (KEGG) and the cluster of orthologous groups of proteins (COG) database (Caspi et al., 2020; Douglas et al., 2020).

### Fecal BAs Analysis

Targeted metabolomics profiling was performed to measure the concentrations of 51 BAs in fecal samples following the protocols recommended by the manufacturer and according to the previously published method (Wang et al., 2019). Briefly, 10 mg of each lyophilized fecal sample from mice was mixed with 50 μL sterile water. After homogenization, the sample was extracted by adding 150 μL of acetonitrile containing isotopically labelled internal standards. The mixture was centrifuged at 12,000 rpm for 15 min at 4°C. The supernatants (50 μL) were transferred to a fresh 96-well plate, and further diluted with 150 μL of a mobile phase mixture (mobile phase B-mobile phase A (50:50, v/v) prior to liquid chromatography-tandem mass spectrometry analysis (LC-TQMS) with 5 μL of injection volume. Fecal BAs profiling was performed using an Acquity ultraperformance liquid chromatography (UPLC) system (Waters Corp., Milford, MA, United States) coupled with a Xevo TQ-S mass spectrometer (Waters Corp., Milford, MA, United States). TargetLynx application manager (Waters Corp., Milford, MA, United States) was used to acquire calibration equations and BAs’ concentration of each sample. MetaboAnalyst (version 5.0) was used to analyze the BAs features in each group and gather statistics. The differences in BAs among groups were assessed by partial least squares discriminant analysis (PLS-DA). The Spearman’s correlation was applied to investigate the associations between fecal BAs and the gut microbiota taxa.

### Statistical Analysis

Data were represented as mean ± standard error of mean (SEM) or mean ± standard deviation (SD), and statistical analysis was performed with either R (version 4.1.1) or GraphPad Prism 8.0.1 (GraphPad, San Diego, CA, United States). Data from multiple groups were assessed for significance using one-way or two-way ANOVA followed by Tukey’s or Dunn’s multiple comparisons test. A *p* < 0.05 was considered to be statistically significant. The significant levels were indicated as follows: ∗*p* < 0.05, ∗∗*p* < 0.01, ∗∗∗*p* < 0.001, ∗∗∗∗*p* < 0.0001.

## Results

### *B. longum* CECT 7894 Improves the Efficacy of IFX for DSS-Induced Colitis

**FIGURE 2:**
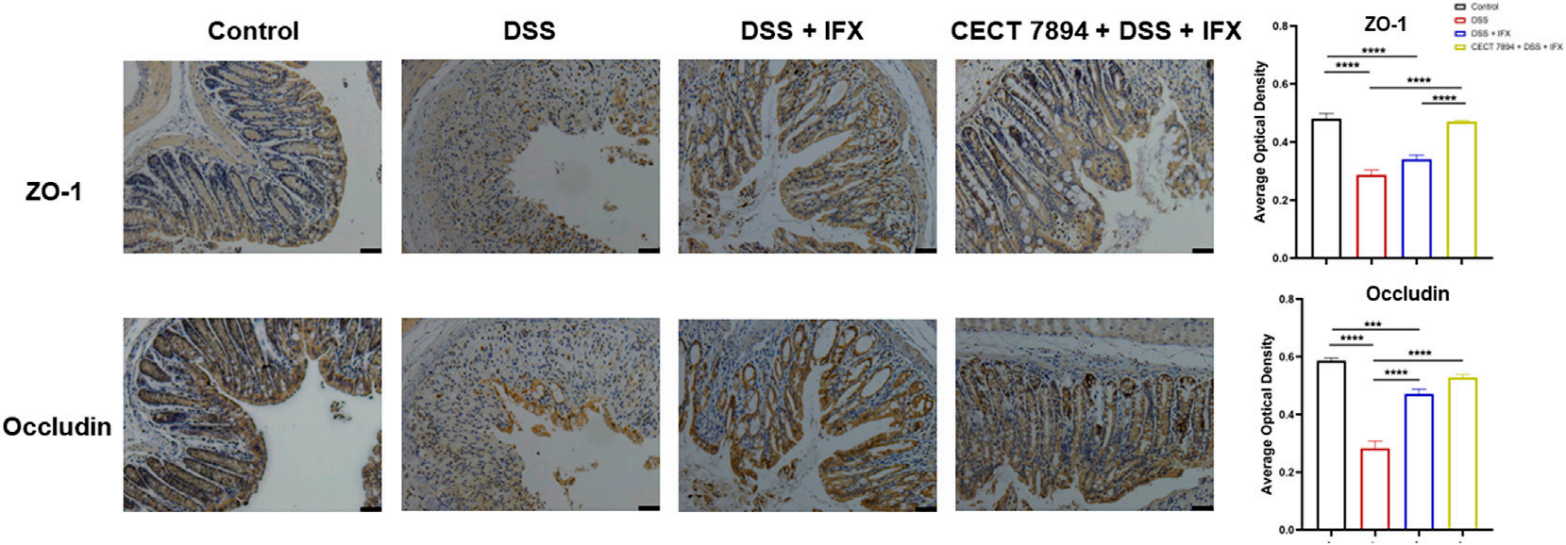
*B. longum* CECT7894 increases tight junction proteins expression. Representative abundances of ZO-1 and Occludin were tested in the colon tissues by immunohistochemical staining (*n* = 6 per group). The scale bar represents 25 µm in each image. Statistical significance was assessed by using one-way ANOVA followed by Tukey’s test. ***p ≤ 0.001, ****p ≤ 0.0001.

To evaluate the effect of *B. longum* CECT 7894 on the efficacy of IFX for DSS-induced colitis, mice were pretreated with *B. longum* CECT 7894 for 5 days prior to DSS administration (Figure 1A). As shown in Figures 1B–D, IFX infusion could significantly attenuate the DSS-induced colitis in mice as evidenced by decreased weight loss, DAI scores, and colon length shortening as compared with the mice received isotype. *B. longum* CECT 7894 supplementation further improved the efficacy of IFX for DSS-induced colitis. In addition, histological assessment by H&E revealed that *B. longum* CECT7894 combined with IFX, maintained better mucus distribution in DSS-treated mice as compared with only IFX, including improved mucosal architecture and goblet cell loss, and reduced inflammatory cell infiltration in the colon tissue (Figure 1E). Furthermore, IHC revealed that *B. longum* CECT7894 combined with IFX treatment increased the expressions of ZO-1 and Occludin in the colon (Figure 2).

### *B. longum* CECT 7894 Modifies Gut Microbiota Composition

**FIGURE 3:**
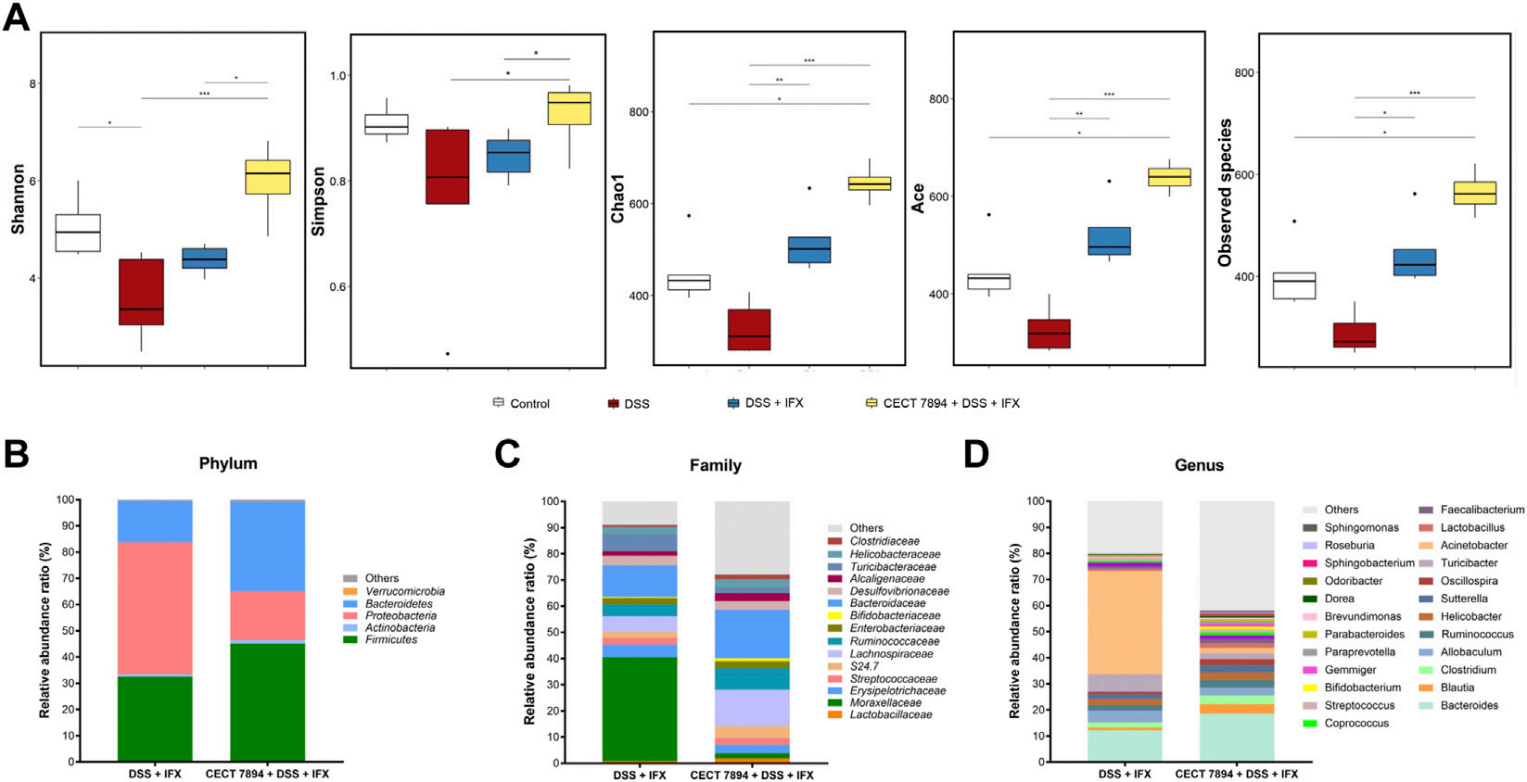
*B. longum* CECT 7894 modulates the gut microbiota diversity and composition. **(A)**The *alpha* diversity evaluated by the Shannon, Simpson, Chao1, ACE, Observed species. **(B)** Relative abundance ratio of bacteria at the phylum levels. **(C)** Relative abundance ratio of bacteria at family levels with the top 15 detected families. **(D)** Relative abundance ratio of bacteria at the genera levels. Statistical significance was assessed by using Kruskal–Wallis H test and the Wilcoxon rank-sum test. *p ≤ 0.05, **p ≤ 0.01, ***p ≤ 0.001.

**FIGURE 4:**
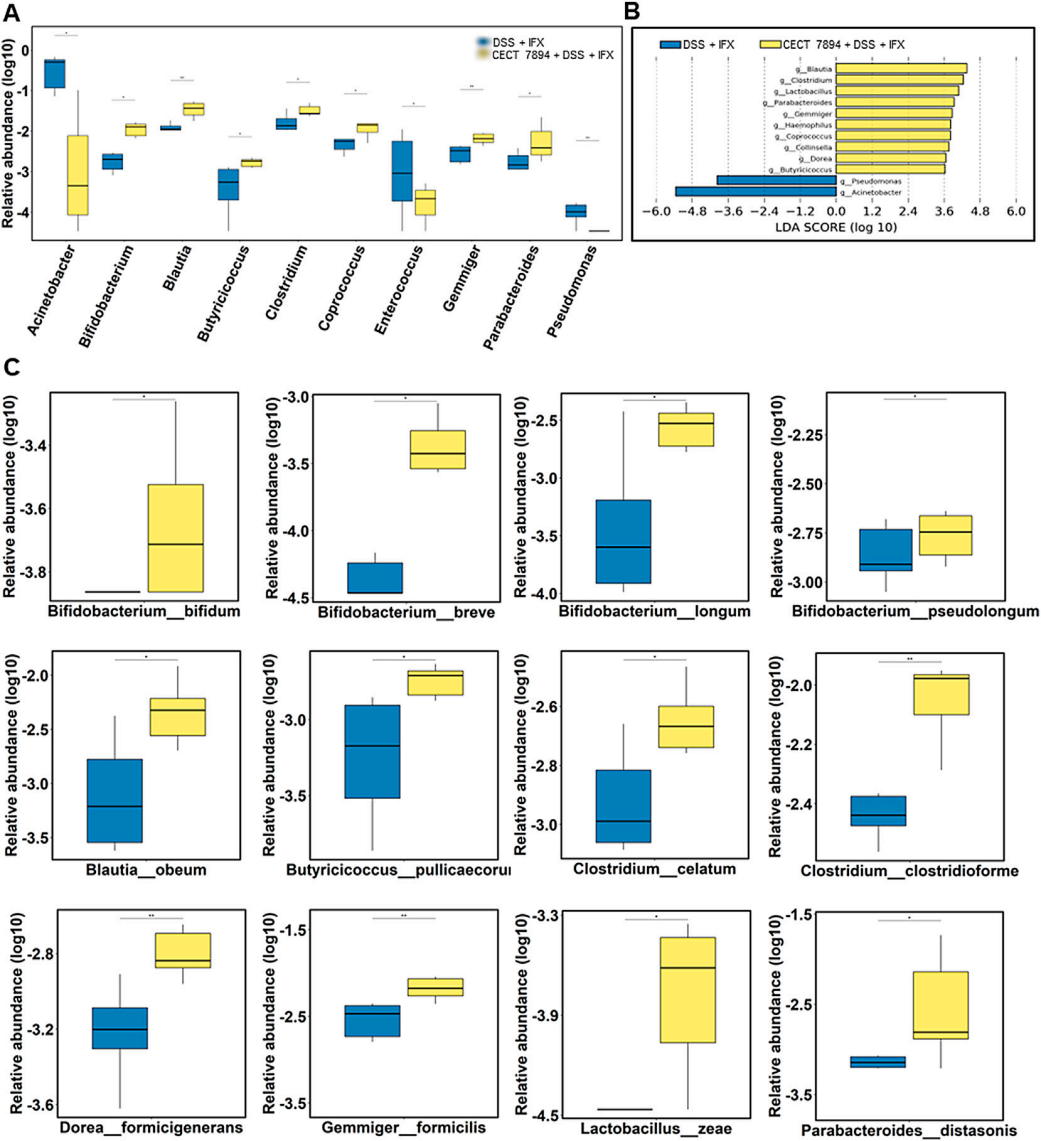
*B. longum* CECT 7894 modifies the gut microbiota composition. **(A)** Relative abundance of bacteria at the genera levels. **(B)** The LEfSe identified the taxa with the greatest differences in the abundance of genera between the DSS + IFX group and *B. longum* CECT 7894 + DSS + IFX group. **(C)** Relative abundance of bacteria at the species levels. Statistical significance was assessed by using Wilcoxon rank-sum test. *p ≤ 0.05, **p ≤ 0.01.

To determine the effect of *B. longum* CECT 7894 on the gut microbiota composition, we performed 16 S rRNA gene sequencing of feces on day 9 after DSS administration. As shown in Figure 3A, the *alpha* diversity indices, including observed species, Shannon, Simpson, Chao1, and ACE were significantly decreased in DSS-treated mice in comparison with the control group, whereas IFX infusion attenuated this effect mediated by DSS treatment. The *alpha* diversity was further increased in *B. longum* CECT + DSS + IFX group. In addition, the *beta* diversity calculated by the PCoA showed a clear separation between the DSS + IFX group and the *B. longum* CECT 7894 + DSS + IFX group (Supplementary Figure S1). Inter-group comparisons of taxonomic profiles showed that the relative abundances of phyla *Firmicutes* and *Bacteroidetes*, families *Bifidobacteriaceae*, *Ruminococcaceae*, *Lachnospiraceas*, *Lactobacillaceae*, and *Bacteroidaceae* were increased, while phyla *Proteobacteria*, families *Moraxellaceae*, *Erysipelotrichaceae*, and *Streptococcaceae* were decreased in *B. longum* CECT 7894 + DSS + IFX group as compared with DSS + IFX group (Figures 3B,C). At the genus level, *B. longum* CECT 7894 supplementation increased the relative abundances of *Bifidobacterium*, *Blautia*, *Butyricicoccus*, *Clostridium*, *Coprococcus*, *Gemmiger*, and *Parabacterioides*, and reduced the relative abundances of *Acinetobacter*, *Enterococcus*, and *Pseudomonas* (Figures 3D,4A). LEfSe analysis further identified the differentially enriched genera between the *B. longum* CECT 7894 + DSS + IFX group and the DSS + IFX group (LDA score >3.5, Figure 4B). Furthermore, the abundance of several bacteria species were increased by *B. longum* CECT 7894 supplementation, particularly members of *Bifidobacterium*, including *B. bifidum*, *B. breve*, *B. longum*, and *B. pseudolongum* (Figure 4C).

### *B. longum* CECT 7894 Changes the Functional Profile of the Gut Microbiome

**FIGURE 5:**
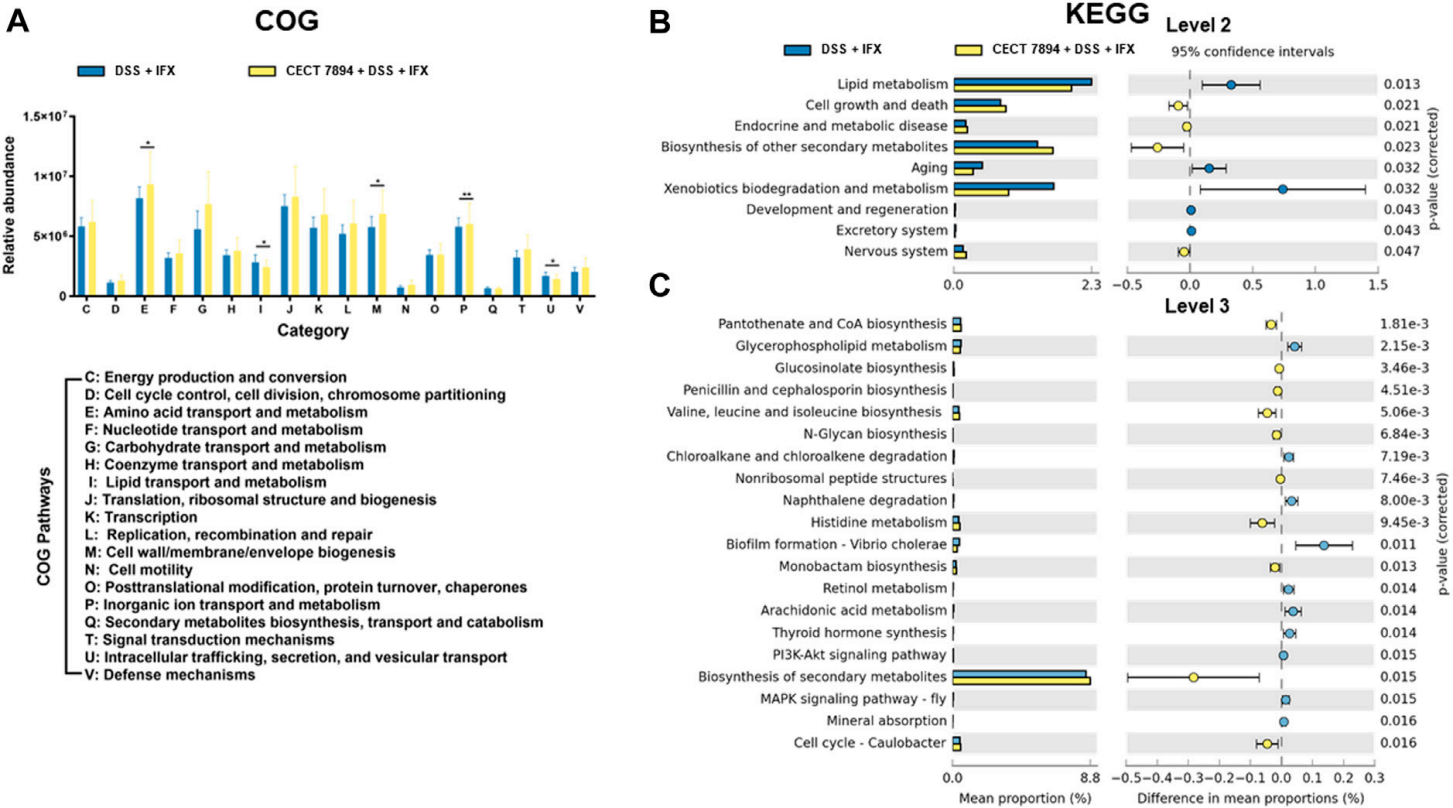
Functional profiles of the gut microbiome. **(A)** Analysis of fecal microbiome on COG pathways. Analysis of the fecal microbiome on KEGG pathways at level 2 **(B)** and level 3 **(C)**. Significance is determined by using the Welch’s t-test, with *p < 0.05, **p ≤ 0.01, ***p ≤ 0.001.

To investigate the effect of *B. longum* CECT 7894 on the functional profile of the gut microbiome, COG and KEGG pathways analysis using PICRUSt were performed in *B. longum* CECT 7894 + DSS + IFX group and DSS + IFX group. The data showed the abundances of COG pathways of energy production and conversion, amino acid transport and metabolism, nucleotide transport and metabolism, carbohydrate transport and metabolism, coenzyme transport and metabolism, translation, ribosomal structure and biogenesis, transcription, replication, recombination and repair, cell wall/membrane/envelope biogenesis, cell motility, posttranslational modification, protein turnover, chaperones, inorganic ion transport and metabolism, secondary metabolites biosynthesis, transport and catabolism, signal transduction mechanisms, and defense mechanisms were higher in *B. longum* CECT 7894 + DSS + IFX group than DSS + IFX group (Figure 5A). Furthermore, similar patterns of metabolic function changes were observed on KEGG analysis. For instance, KEGG pathways of cell growth and death and biosynthesis of secondary metabolites were significantly increased in the *B. longum* CECT 7894 + DSS + IFX group (Figures 5B,C).

### *B. longum* CECT 7894 Modulates the BAs Metabolism

**FIGURE 6:**
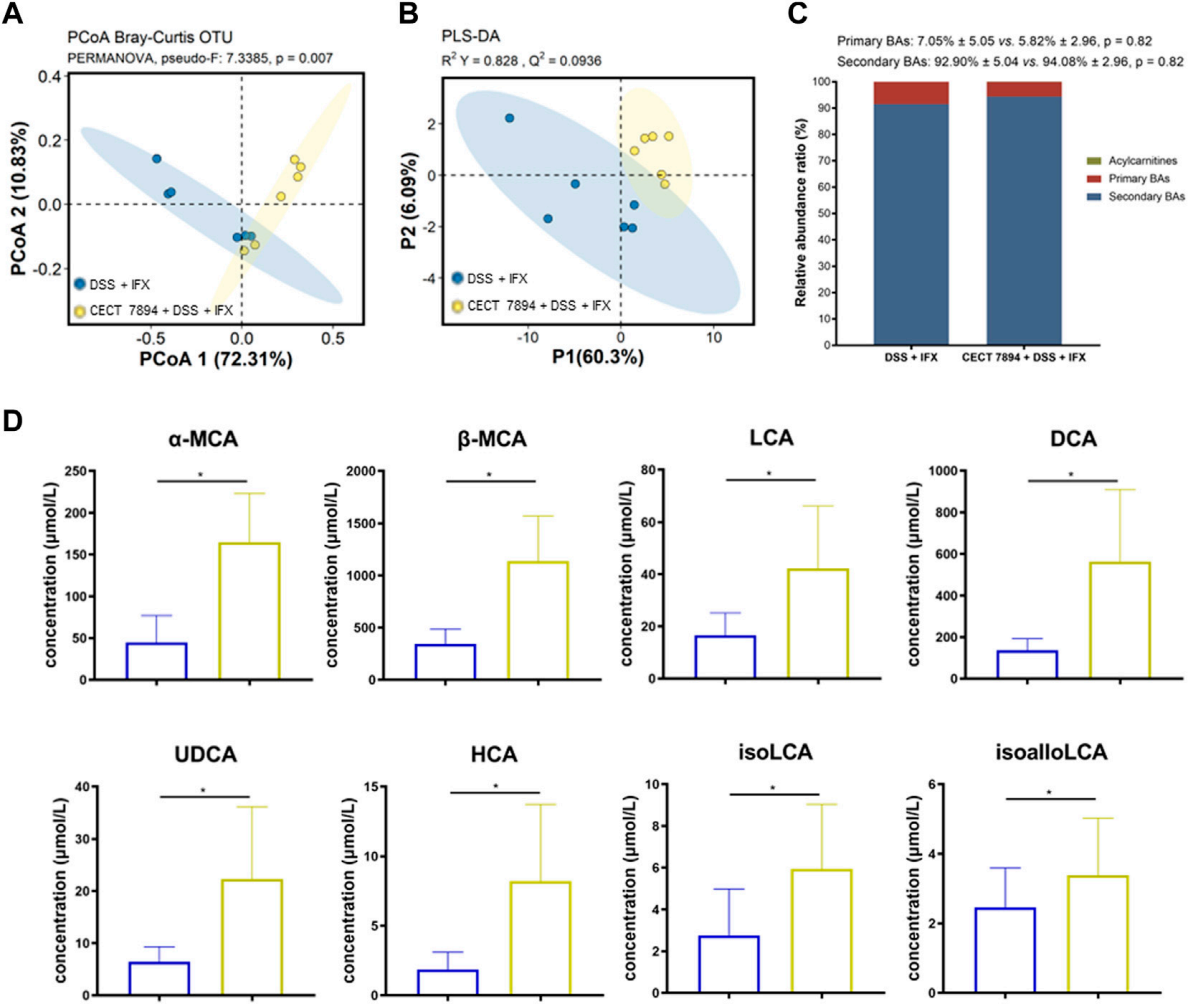
*B. longum* CECT 7894 modulates the fecal BAs levels. **(A)** PCoA plot based on Bray-Curtis distance of fecal BAs between the DSS + IFX group and *B. longum* CECT 7894 + DSS + IFX group. **(B)** PLS-DA models discriminated fecal metabolites between the DSS + IFX group and *B. longum* CECT 7894 + DSS + IFX group. **(C)** Relative abundance ratio of different BAs. **(D)** Bar charts showing representatively changed fecal BAs between the DSS + IFX group and *B. longum* CECT 7894 + DSS + IFX group. Data were presented as mean ± SD. Significance is determined by using the Wilcoxon rank-sum test., with *p < 0.05.

Targeted metabolomics profiling was used to determine the fecal BAs levels in the DSS + IFX group and *B. longum* CECT 7894 + DSS + IFX group (Supplementary Table S1). The PCoA and PLS-DA plots revealed significantly different distribution patterns of BAs between the two groups (Figures 6A,B). The relative abundances of secondary BAs were higher in the *B. longum* CECT 7894 + DSS + IFX group than that of the DSS + IFX group (Figure 6C). Particularly, the concentrations of several secondary BAs, including *a*-Muricholic acid (α-MCA), *ß*-MCA, lithocholic acid (LCA), chenodeoxycholic acid (CDCA), ursodeoxycholic acid (UDCA), hyocholic acid (HCA), isoLCA, and isoalloLCA, were significantly increased in the *B. longum* CECT 7894 + DSS + IFX group (Figure 6D).

### Covariance Between the Gut Microbiota and Fecal BAs

**FIGURE 7:**
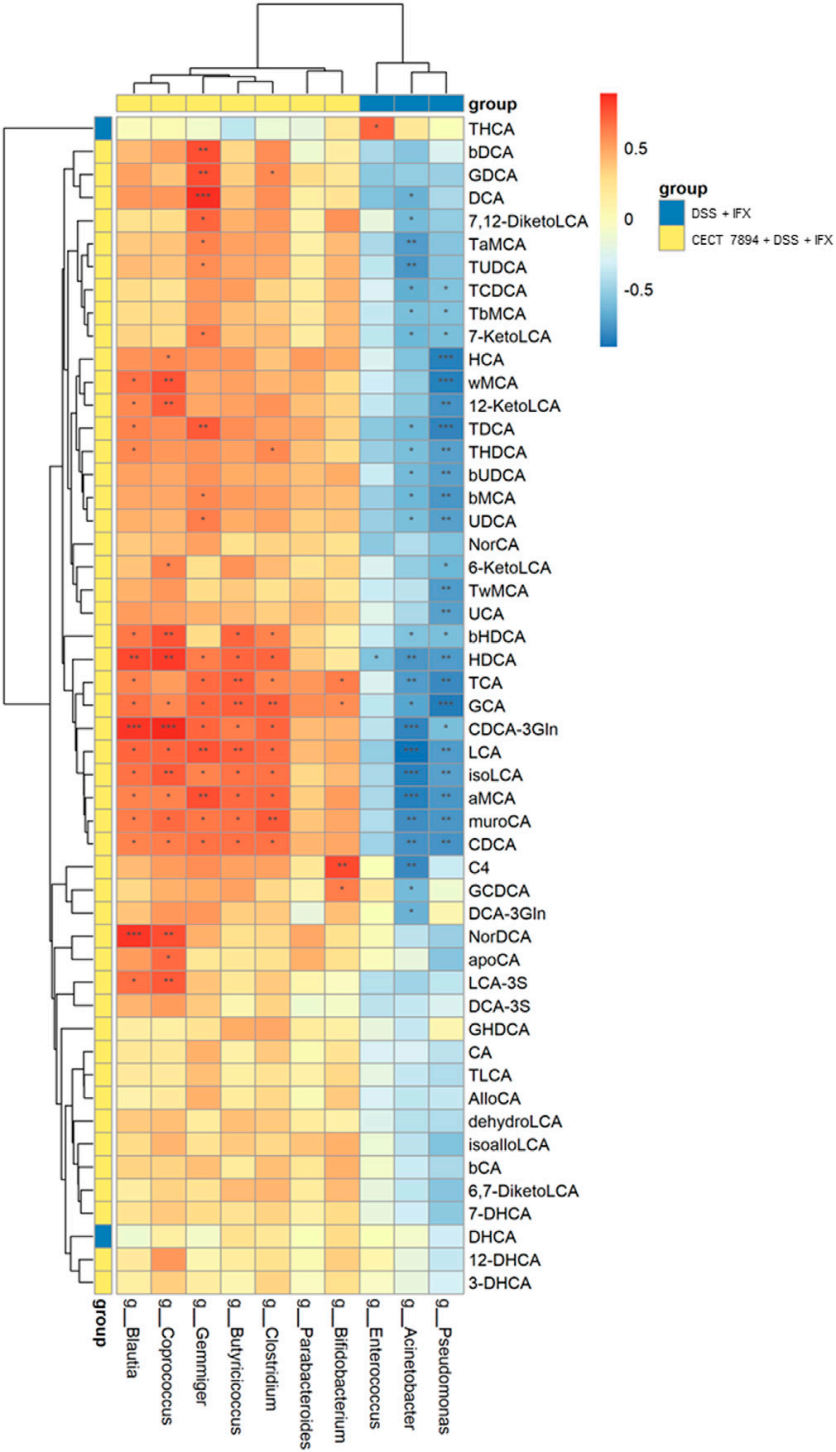
Correlations among the fecal microbiota and fecal BAs. Spearman correlation analysis were performed between top 10 significant differences of bacteria genera and BAs between the DSS + IFX group and *B. longum* CECT 7894 + DSS + IFX group. Blue color represents negative correlations, and red indicates positive correlations. *p < 0.05, **p ≤ 0.01, ***p ≤ 0.001.

To examine the correlations between the bacteria genera and fecal BAs, we performed unsupervised clustering by selecting specific bacteria genera that were modified by *B. longum* CECT 7894 and fecal BAs in the DSS + IFX group and *B. longum* CECT 7894 + DSS + IFX group. As shown in Figure 7, the increased abundances of genera by *B. longum* CECT 7894 were positively correlated with the levels of secondary BAs. For instance, the relative abundances of *Gemmiger*, *Blautia*, *Coprococcus*, *Butyricicoccus*, and *Clostridium* were positively correlated with the increased levels of deoxycholicacid (DCA), hyodeoxycholic acid (HDCA), LCA, isoLCA, aMCA, murocholicacid (muroCA), and CDCA. In contrast, the genera enriched in the DSS + IFX group, including *Acinetobacter*, *Enterococcus*, and *Pseudomonas*, were negatively correlated with the secondary BAs levels as mentioned above.

## Discussion

Anti-TNF therapy has been a milestone in the management of IBD in the past two decades (Torres et al., 2020). Although a high primary response can be achieved in IBD patients treated with anti-TNF agents, the long-term outcome is still suboptimal due to the loss of clinical response (Allez et al., 2010). Thus, it is very important to develop strategies to improve the efficacy of anti-TNF therapy for IBD treatment. The unequal clinical efficacy of different anti-TNF agents between IBD and other chronic inflammatory diseases (e.g., rheumatoid arthritis) suggested that the effector mechanism of anti-TNF therapy in IBD may not be attributed to the TNF blockade alone (Levin et al., 2016). For instance, lamina propria T cell apoptosis and M2 type wound healing macrophage induction have been explored as additional mechanisms of the action of anti-TNF agents in IBD treatment (Atreya et al., 2011; Vos et al., 2011).

Since disturbed gut microbiota is implicated in the IBD pathogenesis, various studies have been focused on the correlations of the gut microbiota composition and IBD, including disease development, and treatment outcome (Glassner et al., 2020; Lavelle and Sokol, 2020). The changes in specific bacteria taxa were reported to be associated with the success of anti-TNF therapy in IBD, such as increased relative abundances of *Bifidobacterium*, *Collinsella*, *Lachnospiraceae*, and *Roseburia* (Rajca et al., 2014; Magnusson et al., 2016; Wang et al., 2018; Yilmaz et al., 2019). Our previous studies showed that IFX sustained response in pediatric CD was positively associated with expansions of *Blautia*, *Faecalibacterium*, and *Lachnospira* (Wang et al., 2018). Although the studies mentioned above revealed the role of gut microbiota composition in predicting the clinical response of anti-TNF therapy in IBD, it remains unclear whether gut bacteria are directly involved in the action of anti-TNF agents. In this study, we showed that a probiotic strain isolated from a healthy child, *B. longum* CECT 7894, enhanced the efficacy of IFX for DSS-induced colitis in mice. Together with the favorable outcome of probiotic strains (e.g. *E. coli* Nissle 1917; probiotic complex VSL#3, and *B. longum* BB536) as mono or adjuvant therapy in IBD from previous clinical studies (Kruis et al., 2004; Saez-Lara et al., 2015; Akutko and Stawarski, 2021), it suggests that microbial-based interventions for restoring the balance of gut microbiota may provide a possibility in improving the clinical response of anti-TNF agents in IBD management.

Next, we attempted to explore the potential mechanism involved in the action of *B. longum* CECT 7894 on the IFX efficacy. It has been reported that the actions of probiotics are major through normalizing the altered gut microbiota (Oka and Sartor, 2020; Akutko and Stawarski, 2021). Indeed, *B. longum* CECT 7894 modified the composition and structure of the gut microbiota community in DSS-induced colitis mice, as revealed by both *alpha* diversity and *beta* diversity analysis. *B. longum* CECT 7894 supplementation increased the relative abundances of several beneficial potential genera, including *Bifidobacterium*, *Blautia*, *Butyricicoccus*, *Clostridium*, *Coprococcus*, *Gemmiger*, and *Parabacterioides,* and reduced potentially pathogeneic bacteria (e.g., *Enterococcus* and *Pseudomonas*). Interestingly, the abundances of specie *B. longum* and several other *Bifidobacterium* species, such as *B. bifidum*, *B. breve*, *B. longum*, and *B. pseudolongum,* were upregulated in the *B. longum* CECT 7894 + DSS + IFX group after discontinuation of *B. longum* CECT 7894 treatment for 9 days. It is likely that *B. longum* CECT 7894 may be able to colonize in the mice gut and create a favorable surrounding for the growth of *Bifidobacterium*. Furthermore, the COG and KEGG pathways analysis revealed that *B. longum* CECT 7894 could change the metabolic activities of the gut microbiota in DSS-treated mice.

Metabolites derived from bacterial metabolism play key roles in the microbiota-host interactions, including BAs (Dorrestein et al., 2014). The gut microbiota metabolizes the BAs by modulating BA synthesis, deconjugation, dehydroxylation, and dehydrogenation (Ridlon et al., 2014). For example, BA deconjugation is catalyzed by BSH, and primary BAs are dehydroxylated into secondary BAs by 7α-dehydroxylases produced by gut bacteria, respectively (Begley et al., 2005). Emerging evidence indicates that altered BAs metabolism correlated with gut microbiota dysbiosis plays a critical role in IBD (Yang M et al., 2021). Previous studies have demonstrated that some secondary BAs, like tauroursodeoxycholic acid (TUDCA), UDCA, and LCA, have protective functions in maintaining intestinal epithelial barrier integrity and attenuating colitis (Laukens et al., 2014; Ward et al., 2017). It was reported that isoalloLCA was significantly reduced in IBD patients, suggesting the important role of isoalloLCA in maintaining intestinal homeostasis (Li et al., 2021). In this study, we found that *B. longum* CECT 7894 changed the BAs metabolism by increasing the abundance of secondary BAs such as *a*-MCA, *ß*-MCA, LCA, CDCA, UDCA, HCA, isoLCA, isoalloLCA. Thus, the increased levels of secondary BAs may be involved in the action of *B. longum* CECT 7894 on the efficacy of IFX for DSS-induced colitis. The covariance analysis further revealed that the levels of secondary BAs were positively associated with the increased abundance of bacteria that contain BSH and 7α-dehydroxylases genes, such as *Bifidobacterium* and *Clostridium*. Furthermore, BAs are endogenous ligands exerting diverse actions through activating specific cell surface and nuclear receptors expressed in the gastrointestinal tract, including FXR, takeda G protein-coupled receptor 5 (TGR5), pregnane X receptor (PXR), and vitamin D receptor (VDR) (Thibaut and Bindels, 2022). It has been shown that FXR activation inhibits inflammation and preserves the intestinal barrier in IBD (Gadaleta et al., 2011). We showed that *B. longum* CECT7894 increased the expression of FXR in the colon tissue as demonstrated by an immunohistochemistry assay (Supplementary Figure S2), which further suggested the role of the BA metabolism in the enhancement of IFX efficacy by *B. longum* CECT7894.

In summary, our data showed that *B. longum* CECT 7894 improved the efficacy of IFX for DSS-induced colitis *via* regulating the gut microbiota and bile acid metabolism. Probiotics supplementation may provide a possibility to improve the clinical response of anti-TNF agents in IBD management.

## Data Availability

The original contributions presented in the study are included in the article/Supplementary Material; further inquiries can be directed to the corresponding authors.
