# Supplementary material for: Bifidobacterium longum CECT 7894 Improves the Efficacy of Infliximab for DSS-Induced Colitis via Regulating the Gut Microbiota and Bile Acid Metabolism
Source: Front Pharmacol. 2022 Aug 1;13:902337. doi: 10.3389/fphar.2022.902337 (PMC9376241; doi:10.3389/fphar.2022.902337)
Supplement: Supplementary file 1 [file Table1.DOCX]

**Supplementary Tables**

**Table S1.** Changes of fecal bile acids between DSS + IFX group and *B. longum* CECT 7894 + DSS + IFX group

| **Class** | **metabolites** | **DSS + IFX group mean** | ***B. longum* CECT 7894 + DSS + IFX group mean** | **p value**^#^ |
| --- | --- | --- | --- | --- |
| Acylcarnitines | C4 | 1.175 | 1.361666667 | 0.108074 |
| Primary BAs | TCA | 2.306666667 | 4.885 | 0.002165 |
|  | TCDCA | 0.311666667 | 0.868333333 | 0.093074 |
|  | CA | 57.52333333 | 299.195 | 0.132035 |
|  | GCA | 0.143333333 | 0.403333333 | 0.004998 |
|  | GCDCA | 0.35 | 0.403333333 | 0.297107 |
|  | CDCA | 6.34 | 24.35666667 | 0.008658 |
|  | CDCA-3Gln | 0.205 | 0.375 | 0.010139 |
| Secondary BAs | TwMCA | 4.986666667 | 9.253333333 | 0.132035 |
|  | TaMCA | 0.386666667 | 1.505 | 0.064935 |
|  | TbMCA | 7.476666667 | 13.60333333 | 0.064935 |
|  | THCA | 0.022 | 0.018833333 | 0.865599 |
|  | TUDCA | 0.296666667 | 0.871666667 | 0.064935 |
|  | THDCA | 0.238333333 | 0.515 | 0.019373 |
|  | TDCA | 0.133333333 | 1.348333333 | 0.0063 |
|  | TLCA | 0.071666667 | 0.181666667 | 0.147401 |
|  | UCA | 0.861666667 | 6.496666667 | 0.025974 |
|  | wMCA | 328.2216667 | 747.8616667 | 0.041126 |
|  | bCA | 1.218333333 | 21.82833333 | 0.093074 |
|  | aMCA | 44.75166667 | 366.535 | 0.004329 |
|  | bMCA | 345.295 | 1115.591667 | 0.015152 |
|  | HCA | 1.865 | 8.208333333 | 0.015152 |
|  | AlloCA | 4.188333333 | 40.93666667 | 0.179654 |
|  | NorCA | 1.79 | 5.866666667 | 0.064935 |
|  | GHDCA | 0.053333333 | 0.058333333 | 0.621014 |
|  | GDCA | 0.101666667 | 0.376666667 | 0.012436 |
|  | muroCA | 16.25333333 | 66.96333333 | 0.015152 |
|  | bUDCA | 0.9 | 3.306666667 | 0.020022 |
|  | bHDCA | 5.131666667 | 7.583333333 | 0.24026 |
|  | UDCA | 6.435 | 28.93 | 0.015152 |
|  | HDCA | 24.23833333 | 46.295 | 0.025974 |
|  | bDCA | 15.11333333 | 40.06 | 0.064935 |
|  | DCA | 136.96 | 563.4416667 | 0.008658 |
|  | NorDCA | 0.686666667 | 2.091666667 | 0.041126 |
|  | isoalloLCA | 2.456666667 | 3.385 | 0.393939 |
|  | isoLCA | 2.758333333 | 5.94 | 0.041126 |
|  | LCA | 16.63333333 | 54.68666667 | 0.008658 |
|  | LCA-3S | 0.136666667 | 0.506666667 | 0.226491 |
|  | dehydroLCA | 15.115 | 17.725 | 0.699134 |
|  | 6-KetoLCA | 11.07666667 | 14.025 | 0.393939 |
|  | 7-KetoLCA | 3.025 | 14.88333333 | 0.041126 |
|  | 12-KetoLCA | 98.77 | 180.1316667 | 0.064935 |
|  | apoCA | 2.808333333 | 3.803333333 | 0.199397 |
|  | 6,7-DiketoLCA | 1.203333333 | 3.753333333 | 0.393939 |
|  | 7,12-DiketoLCA | 0.361666667 | 0.991666667 | 0.044951 |
|  | DHCA | 0.463333333 | 0.421666667 | 0.936075 |
|  | 7-DHCA | 25.665 | 125.7316667 | 0.24026 |
|  | 12-DHCA | 6.636666667 | 21.59333333 | 0.818182 |
|  | 3-DHCA | 6.288333333 | 18.695 | 0.630356 |
|  | DCA-3Gln | 0.213333333 | 0.245 | 0.296258 |
|  | DCA-3S | 0.243333333 | 0.564 | 0.297107 |

^#^The data were compared by the nonparametric Mann-Whitney test.
