# Supplementary figures and images for: Bifidobacterium longum CECT 7894 Improves the Efficacy of Infliximab for DSS-Induced Colitis via Regulating the Gut Microbiota and Bile Acid Metabolism
Source: Front Pharmacol. 2022 Aug 1;13:902337. doi: 10.3389/fphar.2022.902337 (PMC9376241; doi:10.3389/fphar.2022.902337)

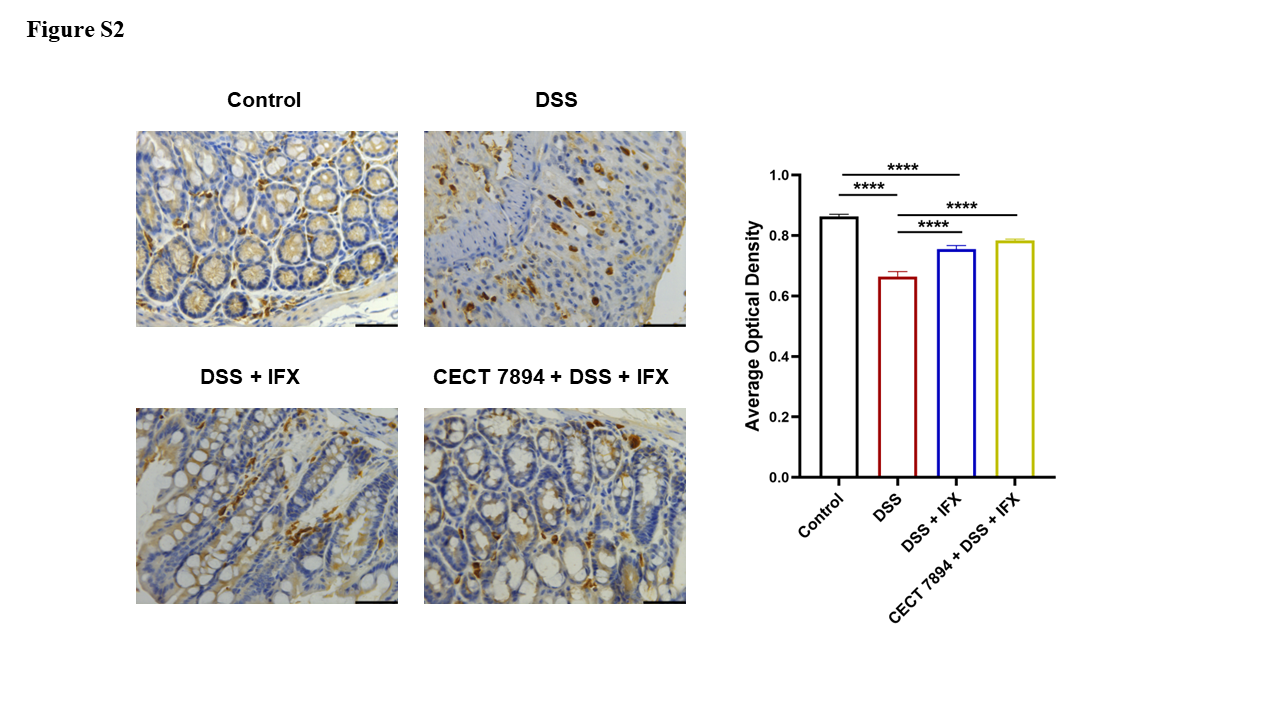

Supplement: Supplementary file 2 [file Image2.TIF]

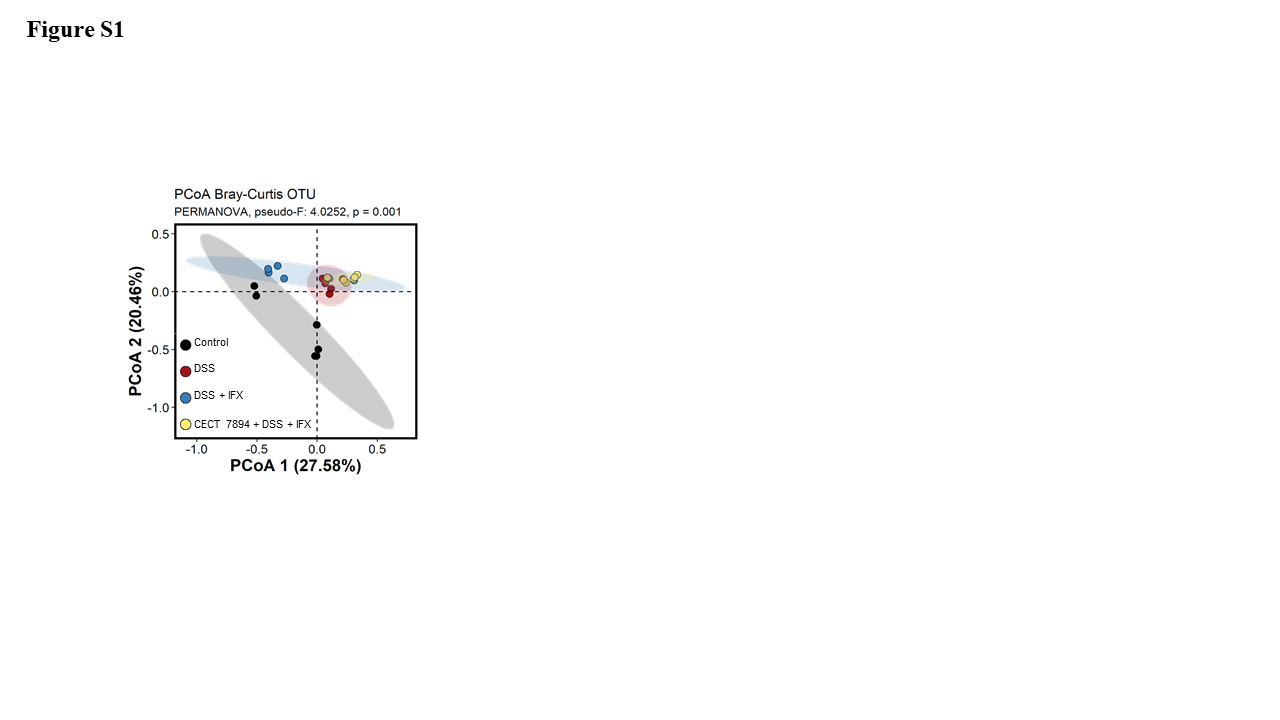

Supplement: Supplementary file 3 [file Image1.TIF]
